# Soybean protein-derived peptide nutriment increases negative nitrogen balance in burn injury-induced inflammatory stress response in aged rats through the modulation of white blood cells and immune factors

**DOI:** 10.29219/fnr.v64.3677

**Published:** 2020-06-29

**Authors:** Jian Zhang, Wenhui Li, Zhiwei Ying, Di Zhao, Guofu Yi, He Li, Xinqi Liu

**Affiliations:** Beijing Advanced Innovation Center for Food Nutrition and Human Health, Beijing Technology and Business University, Beijing, China

**Keywords:** soybean protein-derived peptide, nutritional supplement, negative nitrogen balance, burn, inflammation

## Abstract

**Background:**

As an important nutrient, soybean protein-derived peptides (SPP) affect the immune function of animals.

**Objective:**

This study describes the effects of nutrient supplementation with SPP on the negative nitrogen balance in the burn injury-induced inflammatory response of aged rats.

**Design:**

Soybean protein isolate (SPI) was hydrolyzed to obtain SPP. A negative nitrogen-balance aged rat model and a major full-thickness 30% total body surface area (TBSA) burn-injury rat model were utilized.

**Results:**

The results show that SPP can increase the speed and ability of inflammatory stress by adjusting white blood cell counts. Soybean protein-derived peptides significantly increased serum immunoglobulin M (IgM), immunoglobulin G (IgG) and immunoglobulin A (IgA) levels; significantly decreased serum interleukin-1 beta (IL-β), tumor necrosis factor-alpha (TNF-α) and regulated upon activation normal T-cell expressed and secreted (RANTES) levels. These results give conclusive evidence that SPP has a significantly positive effect in improving the immune function on the condition of negative nitrogen balance with burn-injury, and reducing excessive inflammation.

**Conclusions:**

Nutrient supplementation of SPP can, therefore, be used as an adjuvant treatment to inhibit the development and severity of inflammatory reactions caused by burns, providing a novel therapy for the treatment and positive prognosis of burn patients.

## Popular scientific summary

- Successfully established the negative nitrogen-balance aged rat model.
- Nutrient supplementation with soybean peptides is beneficial in increasing the speed of white blood cells production in rats after burning.
- Soybean peptides advantageously improved the inflammatory stress response ability compared with the soybean protein.
- Nutrient supplementation with soybean peptides offers great potential as a practical and effective intervention for the treatment of severe burn injuries and helps to improve the outcomes for these patients.

The basal metabolism and digestive capacities of the human body decrease with aging, especially in terms of protease activity. The reduced absorption of protein nutrients causes a negative nitrogen balance in the body, which leads to health problems such as muscle atrophy and the decline of immunity (1, 2). It has been reported that aging slows the immune system’s response to injury, delays collagen synthesis, slows the process of wound contraction, and decreases tensile strength in healed tissue (3). The development of low-grade, chronic, systemic inflammation is often observed with aging, characterized by an elevation in inflammatory mediators, which has been termed inflammaging (4, 5). Accompanied by today’s rapid increase in the aging population around the world, these age-related diseases have become important, not only nutritionally but also with regard to socioeconomic concerns.

Inflammation is at the root of virtually every pathology and is associated with many human diseases, including arthritis, obesity, cancer, and atherosclerosis (6, 7). Thus, there is great interest in understanding how inflammation can be regulated and how best to design pharmaceuticals and other interventions to control it. Burns are serious and debilitating injuries and the 10th most common cause of accidental death in children and adults (8). Burns of more than 20% of the body’s total surface area can result in extensive metabolic, inflammatory, endocrine and immune system damage, which can predispose patients to malnutrition, poor wound healing, cachexia, and frequent infections (9). Major burns rapidly induce a significant local inflammatory response and an acute systemic response, which play central roles in the complex dynamics of burn injury-induced muscle atrophy (10). Excessive or prolonged inflammation in burn-injured patients increases their risk for the development of hypermetabolic states and associated muscle loss, shock, and multiple organ dysfunction syndrome. Hypermetabolism leads to malnutrition in burn patients, who need increased amounts of energy from carbohydrates, protein, fat, vitamins and minerals, and malnutrition, in turn, decreases cell-mediated and humoral immune responses, thus increasing the risk of infections and inhibiting wound healing. However, this state can be reversed by re-nutrition. When negative nitrogen balanced elderly people suffer from burns, an overreaction of systemic inflammation and severe muscle atrophy may occur due to their low immune response speed and ability. Despite great advances in medical sciences, the role of nutrition in the treatment of patients with such a critical condition is often ignored.

Nutritional support provides the material basis for immune functioning by providing nutrients or directly participating in the metabolism. It is, therefore, recognized as one of the most significant aspects of care for the burned patient (11). Protein is a major nutrient for burn patients and critical for a healthy immune response. When protein and calorie levels are in deficit, white blood cells (WBCs), antigens, antibodies and immune globulin are reduced. Protein-energy malnutrition is evidenced by poor wound healing, muscle wasting, growth retardation and diminished immunocompetence in the weeks and months after the burn has occurred (11).

Peptides from soybeans have been shown to exhibit a variety of functional properties, including immunomodulatory, antioxidant and hypocholesterolemic effects. Soybean protein has beneficial effects on diabetes, obesity and serum lipids (12–20). Takahiro isolated the immunostimulating peptide His–Cys–Gln–Arg–Pro–Arg from a tryptic digest of soybean proteins, which has homology to tuftsin (Thr–Lys–Pro–Arg), is derived from a soybean glycinin subunit and can activate phagocytosis in human neutrophils (21). Furthermore, it was found to stimulate the production of tumor necrosis factor-alpha (TNF-α) when orally administered to mice. A soy-derived transported tripeptide, Val–Pro–Tyr, has been reported to cross intestinal epithelial cells (IECs), exerting anti-inflammatory activity in vitro in THP-1 macrophage cells as well as in IECs, and also reducing colitis symptoms in mice (22). The protein needs of burn patients with a negative nitrogen balance are increased because of losses through urine and their wounds. It is important that these patients receive adequate nutritional support, especially during the recovery phase. Most vegetable proteins, with the exception of soy, require additional sources of proteins to maximize their effectiveness and amino acid profile. Soy protein is considered a complete protein, as it contains most of the essential amino acids that are found in animal proteins (11) and the nutritional value of soy protein is roughly equivalent to that of high-value animal protein. Nonetheless, there are certain disadvantages that limit the development of isolated soy protein supplements, such as solubility and absorption. Protein catabolism cannot be reversed by increased amino acid availability alone, due to a defect in amino acid transportation (23). Soybean peptides have useful physical functional properties, viz., the facility of digestion and improvement in solubility as compared to soybean protein, and their beneficial amino acid composition makes them a valuable nutritional supplement. Nutritional support with small molecular soybean peptides may help with the palliation of pain and improve cytokine levels in burns, in combination with medications.

This study investigated the effect of soybean protein isolate (SPI) and soybean protein-derived peptides (SPP) as nutritional support for the reduction of excessive systemic inflammatory response, as well as for the improvement of burn injury outcomes. Rat models of negative nitrogen balance and major full-thickness 30% total body surface area (TBSA) burn-injury, with severity classifications in accordance with the American Burn Association, were employed. Six experimental groups were examined: (1) burn-injured rats with a positive nitrogen balance, administered phosphate buffer saline (PBS) supplementation; (2) sham uninjured rats with a negative nitrogen balance, administered PBS supplementation; (3) burn-injured rats with a negative nitrogen balance, administered PBS supplementation; (4) burn-injured rats with a negative nitrogen balance, administered high-dose SPP supplementation; (5) burn-injured rats with a negative nitrogen balance, administered low-dose SPP supplementation; and (6) burn-injured rats with a negative nitrogen balance, administered SPI supplementation. The systemic inflammatory response was evaluated on days 0, 1, 3, 5, 7, 9, 11, and 13 after burn injury, through the assessment of systemic WBC counts and the levels of serum interleukin-1 beta (IL-β), TNF-α, and regulated upon activation normal T-cell expressed and secreted (RANTES). The humoral immunity was evaluated on days 3, 7, and 14 after burn injury, by assessing serum immunoglobulin M (IgM), immunoglobulin G (IgG) and immunoglobulin A (IgA) levels. Burn injury wound area and healing were photographically captured over a 2-week period after burn injury.

## Materials and methods

### Materials

SPI (92% protein) for the preparation of peptides was purchased from Shandong Yuxin Bio-Tech Co., Ltd. (Shandong, China). Neutral and alkaline proteases used for enzymolysis of the SPI were provided by Novozymes (Beijing, China). Rainbow pre-stained protein marker (11–180 kDa) was purchased from Genview (Glenview, USA); Laemmli sample buffer was purchased from Bio-Rad (Hercules, USA); Tris-glycine-SDS, 30% Acrylamide/Bis solution, and urea were purchased from Solarbio (Beijing, China); and ammonium persulfate, β-mercaptoethanol, and tetramethylethylenediamine (TEMED) were purchased from Ameresco (Framingham, USA). Pentobarbital Sodium was purchased from Aikon Biomedicine Co., Ltd. (Jiangsu, China). Rat IgM, IgG, IgA, IL-1β, TNF-α, and RANTES ELISA quantification kits were purchased from Myhalic Biotechnology Co., Ltd (Catalog number: RA20444 for IgM, RA20096 for IgG, RA20442 for IgA, RA20020 for IL-1β, RA20035 for TNF-α, RA20511 for RANTES, Bioswamp, Wuhan, China).

### Preparation of soybean protein-derived peptides

Soybean protein-derived peptides were prepared as previously outlined. SPI was added to water to form a protein content of 10%, followed by digestion with two kinds of selective commercial proteases. The proteases were added at the level of 1% of SPI weight, and hydrolysis was carried out at 50°C for 4 h. The digest was heated at 85°C for 15 min to deactivate the enzyme. Thereafter, using the combination of an ultrafiltration membrane and metal film separation, peptides were separated from the enzyme hydrolysis solution of soybean proteins. After sterilization and spray drying, the peptides were prepared as fine, light-yellow granules with faster solvability and better solubility in comparison to soybean proteins.

### SDS-PAGE

To monitor the pattern of SPP, SDS-PAGE was performed on the basis of the method outlined by Laemmli, with slight modifications. Each sample was dissolved in distilled deionized water (DDW) (~2.67 mg protein/mL) containing 480 mg/mL urea, and an aliquot (300 μL) was mixed 3:1 (v/v) with the Laemmli buffer under reducing conditions (0.71 M-mercaptoethanol) and boiled for 5 min. A Rainbow pre-stained protein marker, SPI, enzymatic hydrolysate, and SPP (10 μL, containing ~20 μg protein) were loaded onto 18-well hand-cast 12% acrylamide and 5% stacking gels. The gels were electrophoresed at a constant voltage of 100 V for approximately 1.5 h, then stained using Coomassie Brilliant Blue for another 2 h, followed by destaining. A Molecular Imager Gel Dox XR system (Bio-Rad Laboratories) was used to scan the gels.

### Molecular weight distribution

The distribution of the molecular weight of SPP was analyzed by using a 1260 Infinity II LC system with a TSK-GEL G2000SWXL (5 μm, 7.8 × 300 mm^2^) column. Soybean protein-derived peptides were prepared to a concentration of 1 mg/mL. The samples and standards were all filtered through a 0.22-μm filter membrane with a 200-mesh screen before injection. Soybean protein-derived peptides were separated into an isocratic elution with water/acetonitrile/trifluoroacetic acid (45:55:0.1). The flow rate was set at 0.5 mL/min and the detection wavelength was 220 nm. The injection volume was 10 µL.

### Experimental animals and treatments

All studies adhered to procedures consistent with the International Guiding Principles for Biomedical Research Involving Animals issued by the Council for International Organizations of Medical Sciences (CIOMS) and were approved by the Institutional Animal Care and Use Committee at the First Affiliated Hospital of PLA General Hospital (R18FO10032). A total of 48 eight-month-old male Wistar rats (400–600 g) from Beijing Vital River Laboratory Animal Technology Co., Ltd. were housed in individual stainless steel cages in an air-conditioned room (temperature, 22–24°C; relative humidity, 55–60%; lights on 7:00–19:00). In order to prepare the negative nitrogen-balance aged rat model, the rats were divided into two groups: (1) positive nitrogen balance group (20% casein, *n* = 8) and (2) negative nitrogen balance group (1.5% casein, *n* = 40). The positive nitrogen balance group received an AIN-93G diet containing 20% casein, while the negative nitrogen balance group received the same diet but with only 1.5% casein. The rats were given free access to their food and deionized water for 2 weeks in preparation of the negative nitrogen-balance aged rat model.

**Fig. 1:**
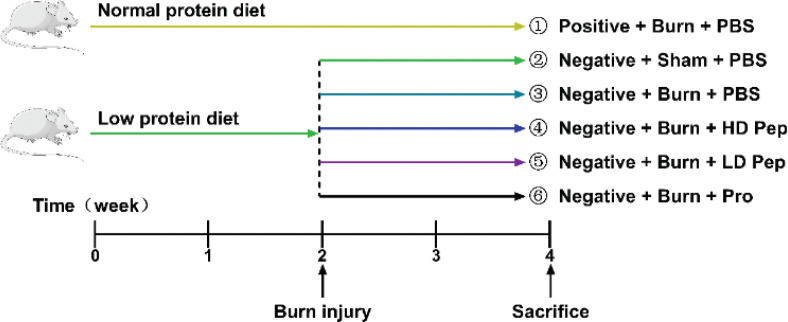
Aged rats and experimental schedule.

After 2 weeks, the rats were randomly divided into the following six groups, each comprising eight mice as shown in Fig. 1: (1) Positive nitrogen + burn injury + PBS supplementation; (2) negative nitrogen + sham injury + PBS supplementation; (3) negative nitrogen + burn injury + PBS supplementation; (4) negative nitrogen + burn injury + high-dose SPP supplementation; (5) negative nitrogen + burn injury + low-dose SPP supplementation; (6) negative nitrogen + burn injury + SPI supplementation. Rats were anesthetized by using intraperitoneal injection of 37.5 mg/kg body weight of 1.5% pentobarbital sodium. Dorsal rat hairs were shaved by using an electric razor. The 30% TBSA thermal full-thickness third-degree burn injury model has previously been established and described (24). In brief, the back skins of the sham injury rat group 2 were placed in water at 37°C for 12 s. The back skins of the burn injury rat groups (1, 3, 4, 5, and 6) were placed in 94˚C water for 12 s. Immediately following injury, a balanced salt solution injection (40 mL/kg body weight) was administered to prevent shock and a 1% tincture of iodine treatment was applied to the burn area to prevent infection. The burn-injured area was left open. All experiments were conducted according to the procedure shown in Fig. 1.

### Dosage information

Rats in groups 1, 2, and 3 were intragastrically administered 3 mL 1× PBS once a day, while those in group 4 were intragastrically administered high-dose SPP (900 mg/kg body weight) constituting 3 mL 1× PBS, those in group 5 were intragastrically administered low-dose SPP (450 mg/kg body weight) constituting 3 mL 1× PBS, and the rats in group 6 were intragastrically administered SPI (450 mg/kg body weight) constituting 3 mL 1× PBS. The doses of SPP and SPI used in the experiment, which were the equivalent of approximately 10 g per day in humans, were based on previous research (2, 25).

### Negative nitrogen-balance rat model analysis

During the period of preparing the negative nitrogen-balance model, rats from each group were weighed on days 1 and 14. Eight mice from each group were housed in the metabolic chambers for 24 h. Fecal and urine samples from the rats were collected and food intake was recorded. The Kjeldahl method was used to measure nitrogen content of food intake, feces and urine. The nitrogen balance of each rat was calculated according to the following formula: nitrogen balance/mg = (intake nitrogen/mg – fecal nitrogen/mg) – urine nitrogen/mg. After feeding for 2 weeks, blood was taken from the tail tips of eight rats from each group. The blood sample was placed in a sterile centrifuge tube, placed at room temperature for about 30 min, centrifuged at 3,000 r/min for 15 min, and the supernatant was separated to obtain serum. Rat serum total protein (TP) and serum albumin (ALB) levels were measured by ELISA, following the manufacturer’s instructions.

### White blood cell count

The tails from the rats in each group were cut on days 0, 1, 3, 5, 7, 9, 11, and 13 after injury protocol. Approximately 100 μL of blood from each animal was collected in a separate heparin-containing separating gel tube, and thoroughly mixed to prevent clotting. The total number of WBCs in the blood was measured by microscopic counting.

### Immunoglobulin measurement

The tails from the rats in each group were cut on days 3, 7, and 14 after injury protocol and approximately 500 μL of blood from each animal was collected in a separate sterilized centrifuge tube. The blood samples were centrifuged, and the supernatant was collected for the detection of serums IgM, IgG, and IgA, which were measured using commercial rat ELISA quantification kits, according to the manufacturer’s instructions. The rats were sacrificed and the spleen and thymus tissue were weighed on day 14.

### Inflammatory cytokine measurements

The tails from the rats in each group were cut on days 3, 7, and 14 after injury protocol and approximately 500 μL of blood from each animal was collected in separate sterilized centrifuge tubes. The blood samples were centrifuged and the supernatant was collected to detect the concentrations of serums IL-1β, TNF-α, and RANTES, which were measured using commercial rat ELISA quantification kits, according to the manufacturer’s instructions.

### Macroscopic evaluation of the wounds

To evaluate the macroscopic wound healing features, the wound area was photographed using a digital camera on days 3, 5, 7, 9, 11, and 13 after the injury protocol.

### Statistical analysis

Data were expressed as the mean ± standard deviation (SD). The results were analyzed using a one-way analysis of variance (ANOVA) followed by Duncan’s multiple range test using SPSS 23 software, and graphs were made by using Graph Pad Prism 7 software. Values of *P* < 0.05 were considered significant.

## Results

### Electrophoresis patterns

**Fig. 2:**
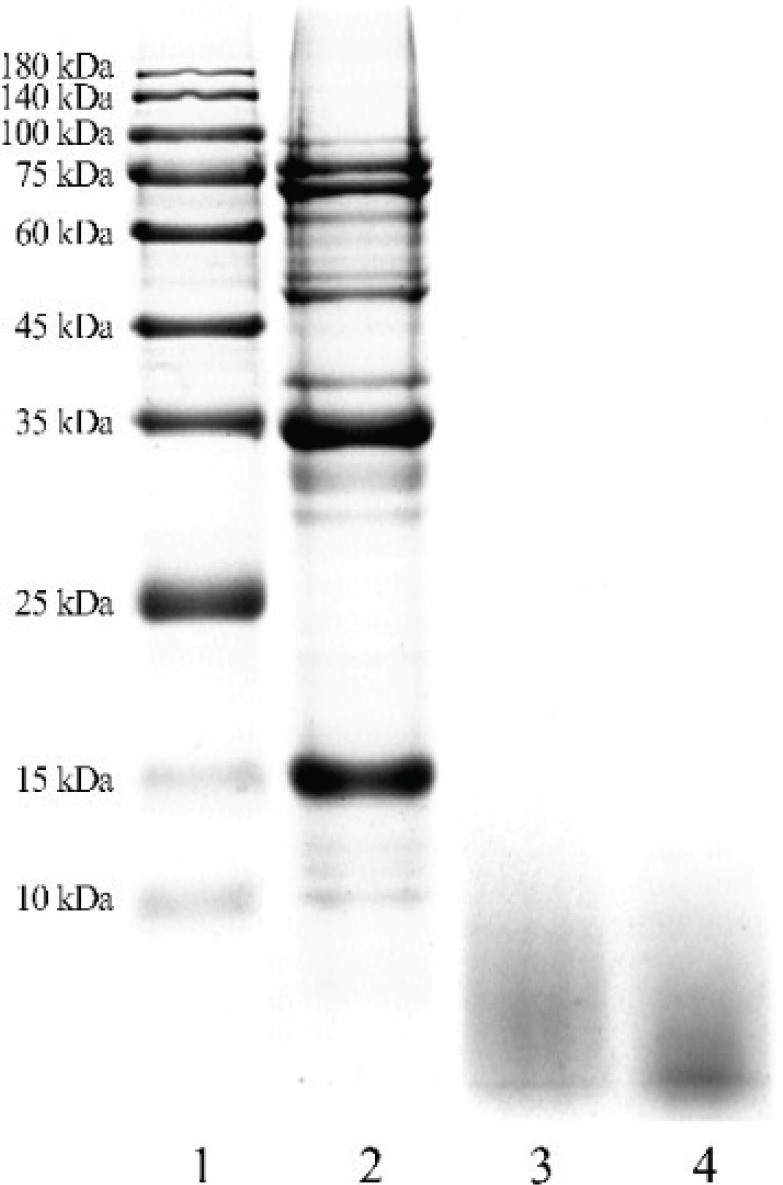
SDS-PAGE visualization of SPI hydrolysis pattern. The amount of protein loaded in each lane is ~20 μg. Lanes: 1, pre-stained protein marker; 2, SPI; 3, enzymatic hydrolysate; 4, SPP.

The electrophoresis patterns of pre-stained protein marker, SPI, enzymatic hydrolysate, and SPP are shown in Fig. 2, where it is evident that there were much high levels of molecular weight proteins in the SPI. The molecular weight of the main peptides in the enzymatic hydrolysate was less than 10 kDa, indicating that most of the SPI had been digested into the peptides with a lower molecular weight. After ultrafiltration membrane and metal film separation, the high molecular weight proteins were removed, leaving the peptides with a higher purity. The electrophoresis patterns of peptides showed that the molecular weight was much lower than SPI and concentrated in a very small range without good separation. This was subsequently further determined by gel filtration chromatography (GFC), as described in the next section.

### Molecular weight distribution

**Fig. 3:**
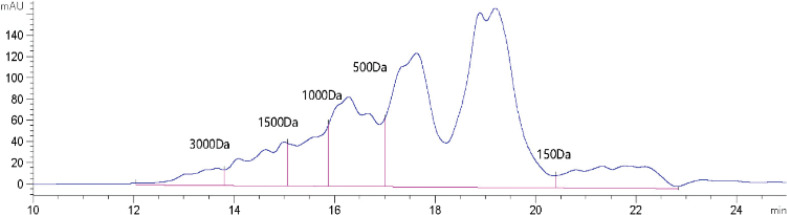
Molecular weight distribution of peptides. Standards were thymosin α1, bacitracin, Gly–Gly–Tyr–Arg, and Gly–Gly–Gly, respectively.

Bioactive peptides generally correspond to low molecular weight peptides (26, 27). The molecular weight of peptides affects their physiological and functional properties. On the basis of the SDS-PAGE results, an Agilent 1,260 Infinity II LC System was used to perform GFC to further determine the molecular weight distribution of peptides. As shown in Fig. 3 and Table 1, peptides with molecular weights of 1,000 Da or less accounted for 82.91% and peptides with molecular weights of 3,000 or more accounted for just 2.87%. The molecular weight distribution was relatively narrow and the molecular weights were much smaller than SPI, in accordance with the results shown in Fig. 2.

**Table 1:** Distributions of molecular weight in soybean protein-derived peptides

Molecular weight (Da)	Integral area (%)	Proportion (%)
>3,000	2.87	2.87
1,500–3,000	7.05	7.05
1,000–1,500	7.15	7.15
150–1,000	75.24	82.91
<150	7.67	

### Negative nitrogen-balance aged rat model

Failure to absorb exogenous protein nutrients could lead to the suppression of protein synthesis and excessive decomposition of the muscle tissue or other protein tissues. Here, the rats had access to the low protein diets for 2 weeks in preparation of the negative nitrogen-balance aged rat model. Nitrogen balance is the most commonly used and most effective indicator for evaluating protein metabolism. A change in body weight is one of the important physiological indicators of negative nitrogen balance. TP and ALB could represent protein metabolism and nutritional status and (28), therefore, the nitrogen metabolism, body weight, and concentrations of TP and ALB serum were measured.

Table 2 shows the nitrogen metabolism, body weight, and concentrations of TP and ALB. The body weight of rats fed on low protein diet was significantly lower on day 14 than on day 1, while the body weight of rats fed a normal protein diet showed no significant change. The concentrations of TP and ALB in serum tended to be lower on day 14 in the rats fed the low protein diet than those fed the normal protein diet, as did their fecal nitrogen and urine nitrogen levels. Thus, the negative nitrogen-balance rat model was considered to have been successfully established.

**Table 2:** Nitrogen metabolism of rats with different diets

	Positive nitrogen balance group	Negative nitrogen balance group
Initial BW (g)	612.6 ± 40.7^a^	611.1 ± 30.9^a^
Final BW (g)	614.7 ± 40.3^a^	574.8 ± 33.0^a^
Nitrogen intake (mg/d)	861.7 ± 87.8^a^	40.9 ± 19.5^b^
Fecal nitrogen (mg/d)	199.8 ± 53.9^a^	30.7 ± 12.1^b^
Urine nitrogen (mg/d)	430.3 ± 64.7^a^	68.2 ± 9.9^b^
Nitrogen balance (mg/d)	231.7 ± 95.3^a^	-58.0 ± 19.5^b^
Total protein (g/L)	74.2 ± 6.5^a^	60.3 ± 5.7^b^
Serum albumin (g/L)	47.6 ± 8.8^a^	39.7 ± 3.0^b^

Data are shown as mean ± SD for eight rats. Means in the same row not sharing a common superscript are significantly different at *P* < 0.05. Positive nitrogen balance groups were fed diets supplemented with 20% casein; negative nitrogen balance group were fed diets supplemented with 1.5% casein for 2 weeks. Nitrogen balance/mg = (intake nitrogen/mg – fecal nitrogen/mg) – urine nitrogen/mg.

### Effect of scald on the total number of white blood cells in rats

**Fig. 4:**
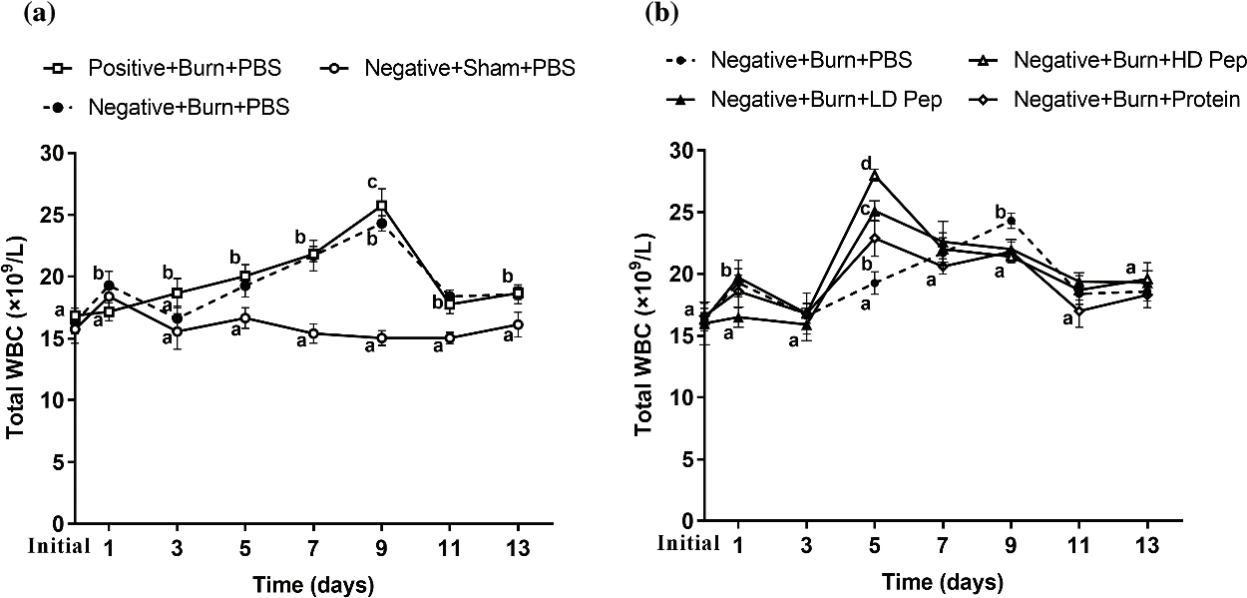
Effects of burn on total white blood cells from aged rats (a). Effects of protein nutrition supplementation on total white blood cells from burn injury aged rats with negative nitrogen balance (b). Blood was obtained from the tail tip. Data are shown as mean ± SD for eight rats. Superscript numbers a, b, c, d designate groups in which statistically significant differences (*P* < 0.05) for each time-point were observed.

As one of the crucial immune cells, leukocytes may increase in total number to resist external infections and exert non-specific immune functions when the body is stimulated by infection or inflammation. Fig. 4(a) shows the effect of the burn on the total number of WBC in the rats. The injection of sodium pentobarbital could have increased the total number of WBC in a short period of time and, thus, the total WBC count in each group was increased slightly on the first day of burn. Leukocytosis was observed following the burn injury, with a significant increase in the WBC count in the burn-injured rats compared with that in the sham rats. After the burn injury, a significantly higher production of leukocytes was measured in both the positive nitrogen balanced group and negative nitrogen balanced group, peaking at day 9. The total number of leukocytes increased faster in the positive nitrogen group than in the negative nitrogen group on the third day after burn. These results indicate that the burn treatment could significantly increase the production of WBCs in aged rats. The WBCs production was stronger in the positive nitrogen rats than it was in the negative nitrogen balanced rats.

Fig. 4(b) shows the effect of protein nutrient supplementation on the total number of WBC in the negative nitrogen balanced rats after burning. Following protein nutrient supplementation, the production of leukocytes was significantly increased in the negative nitrogen balanced rats administered intact protein and peptides than in those rats administered PBS, peaking at day 5. The production speed was faster and number of leukocytes in the high-dose SPP group was significantly higher than those in the low-dose SPP group, while the production speed was slowest and number of leukocytes lowest in the protein group. These results indicate that both protein and peptide nutrient supplementation are beneficial in increasing the speed of WBCs production in rats after burning. The supplementation of protein source nutrients promoted the production of protein immune substances and enhanced the ability of the immune system, while nutrient supplementation with high-dose SPP was determined to be better than low-dose SPP in improving leukocyte production and increasing inflammatory stress response ability. Soybean protein-derived peptides advantageously improved the inflammatory stress response ability compared with the SPI.

### Effect of soybean protein-derived peptide on immune function in rats

**Fig. 5:**
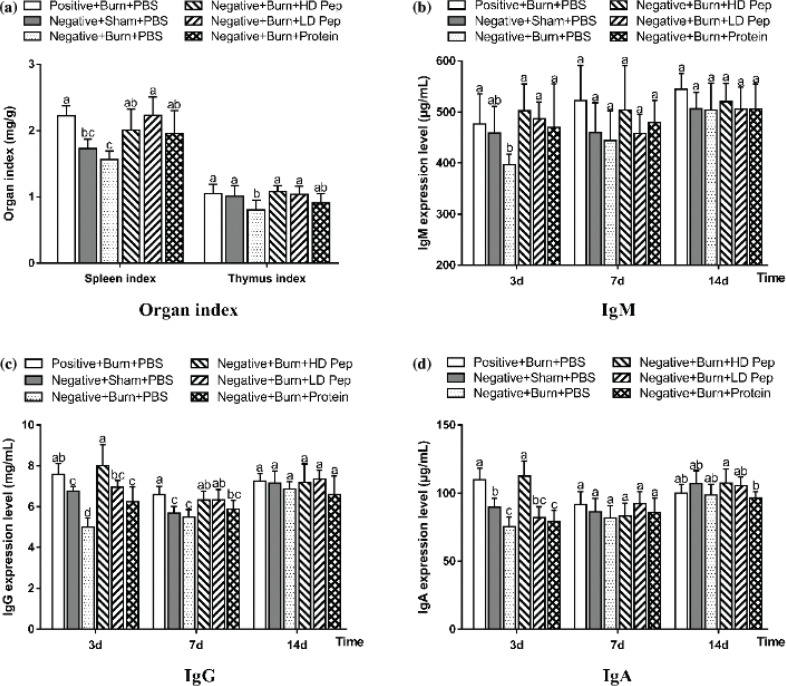
Effects of soybean protein-derived peptides on immune organs index (a) and levels of IgM (b), IgG (c), and IgA (d) in rats. Organs samples were collected from each rats euthanized on day 14. Data are shown as mean ± SD for eight rats. Values not sharing a common letter are significantly different at *P* < 0.05.

The body’s immune function can be assessed by the weight of the immune organs. The rats were sacrificed on day 14, after which their spleen and thymus tissue were weighed. Fig. 5(a) shows the spleen index and thymus index of the rats, of which the indexes of the positive nitrogen rats administered PBS after burn were significantly higher than those of the negative nitrogen rats administered PBS, indicating that the negative nitrogen balance led to the suppression of spleen and thymus growth. The spleen and thymus indexes of the negative nitrogen rats administered SPP were significantly higher than those of the negative nitrogen rats administered PBS. The spleen index values of the negative nitrogen rats administered SPI were significantly higher than those of the negative nitrogen rats administered PBS, while there were no significant differences in the thymus index between the two groups. These results indicate that soybean peptides supplementation had a significant effect on the growth of both spleen and thymus in rats with a negative nitrogen balance after the burn.

Fig. 5(b), 5(c), and 5(d) show comparisons in the serum immunoglobulin on days 3, 7, and 14 following either sham or burn injury in the PBS, SPP, and SPI administered rat groups. The serum IgM, IgG, and IgA levels were markedly lower in the negative nitrogen rats administered PBS than those in the positive nitrogen rats administered PBS at day 3, which indicates that the negative nitrogen balance resulted in the suppression of antibody synthesis. Burn injury resulted in the production of significantly lower IgG and IgA levels on day 3 following the burn injury as compared to those in the sham treatment groups administered PBS, thus indicating that the burn led to the suppression of antibody synthesis. Both IgM and IgG levels were markedly higher in the negative nitrogen rats administered SPP and SPI than in the negative nitrogen rats administered PBS on day 3, thus indicating that soybean peptides and protein nutrient supplementation had a significant promotional effect on the production of antibodies. The serum IgG and IgA levels were higher in the rats administered low-dose SPP than those in the rats administered SPI on days 3, 7, and 14, which indicates that the nutrient supplement of soybean peptides had a better effect on the enhancement of humoral immunity than that of soybean protein.

### Effect of soybean protein-derived peptide on inflammation in rats

**Fig. 6:**
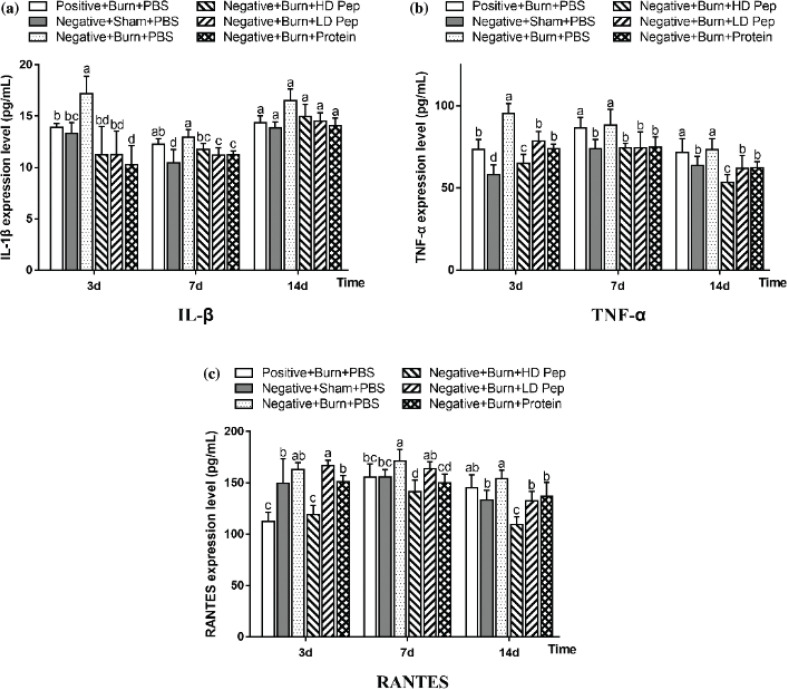
Effects of soybean protein-derived peptides on the levels of Inflammatory factor IL-1β (a), TNF-α (b), and RANTES (c) in rats. Data are shown as mean ± SD for eight rats. Values not sharing a common letter are significantly different at *P* < 0.05.

Inflammatory response is an important pathophysiological change after the body has suffered burn injury. The burn wound healing process is controlled and regulated by various cytokines. Wound tissue cells and inflammatory cells release a large amount of inflammatory factors in response to a burn; however, a high concentration of cytokines could destroy the immune function and lead to an excessive systemic inflammatory response (6). Studies have shown that the levels of pro-inflammatory cytokines IL-1β and TNF-α increase sharply in the inflammatory process and are, thus, regarded as indicators of inflammation (29–31). These cytokines play a vital role in the host’s immune response against infections and inflammatory diseases (32). Here, as shown in Fig. 6(a) and 6(b), the burn injury resulted in the production of significantly higher IL-1β and TNF-α levels on days 3 and 7 as compared to those of the sham treatment groups administered PBS. The IL-1β and TNF-α levels of the negative nitrogen rats administered high-dose SPP, low-dose SPP, and SPI were all significantly lower on days 3 and 7 than those of the negative nitrogen rats administered PBS, thus indicating that protein nutrient supplementation led to the suppression of IL-1β and TNF-α levels in the rats after the burn. As shown in Fig. 6(c), the RANTES levels of the negative nitrogen rats administered high-dose SPP were significantly lower on days 3, 7, and 14 than those of the negative nitrogen rats administered PBS, thus indicating that peptides nutrient supplementation led to the significant suppression of RANTES expression.

### Effect of soybean protein-derived peptide on wound healing in rats

**Fig. 7:**
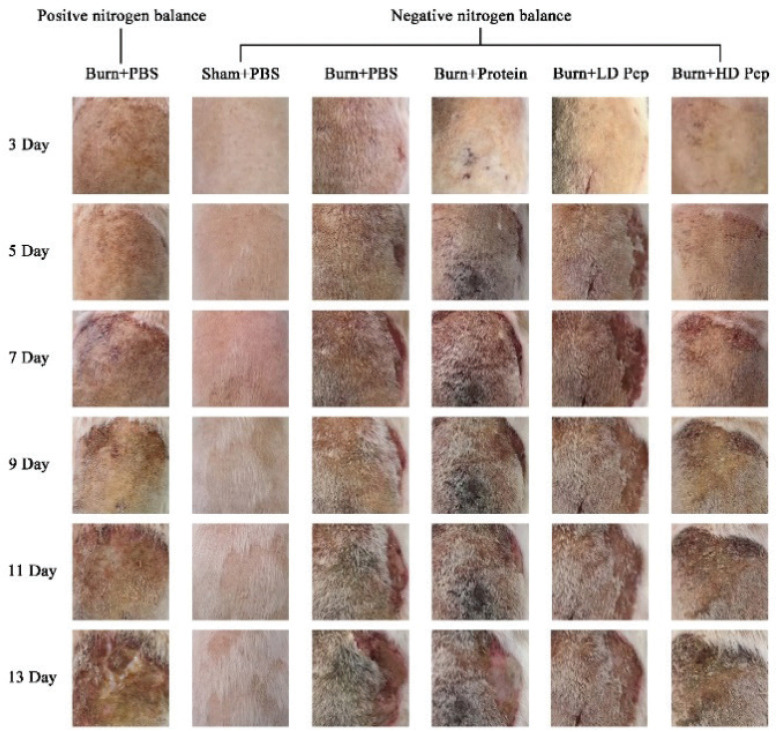
Protein supplementation promotes burn injury wound healing. Injury area and wounds resulting from 30% TBSA burn injury were photographically captured in rats 3, 5, 7, 9, 11, and 13 days post-burn injury.

Optical photographs of the wound area were taken on days 3, 5, 7, 9, 11, and 13. During the first 3 days, the appearance of the wound did not exhibit macroscopic differences between any of the groups. Scabs appeared on the burn wounds on day 3, which did not present differently among the burn groups (Fig. 7). On day 5, the scabs in the SPP group were gradually shedding, while no obvious differences were evident in the other groups. The exudate still appeared in the wounds of the PBS and SPI groups on day 13, while the wound healing of the burn-injured rats that were administered SPP displayed significant improvement compared to that of burn-injured rats administered SPI and PBS.

## Discussion

The above data provides evidence that supplementation with the protein nutrient increased the speed of immune stress and reduced the systemic inflammatory responses induced by major 30% TBSA full-thickness burn injury in the negative nitrogen-balance rat model. The effect of dietary supplementation of soybean protein-derived small molecular weight peptides was superior to that of soybean protein due to its excellent solubility and fast absorption.

With aging, the activities of various proteases in the elderly gradually decline. The reduced digestion and absorption of protein nutrients may result in a negative nitrogen balance in the body, which, in turn, reduces the body’s immune ability and weakens the inflammatory stress response. In this study, the negative nitrogen-balance aged rat model was prepared by feeding with a low protein diet for 2 weeks, during which muscle was consumed to counter the insufficient level of protein in order to maintain normal life activities. This high degradation rate of body protein resulted in a negative nitrogen balance in the body, along with weight loss and muscle atrophy. The inadequate intake of protein nutrients also gave rise to a significant reduction in the concentrations of TP and ALB.

A successful immune response relies on the efficient activation of host defense mechanisms (33). Major burn injury induces a profound inflammatory response and, in severe cases, can lead to multiple organ failure or death. Burns can cause malnutrition and hypermetabolism. Here, the insufficient supply of protein nutrients in rats with a negative nitrogen balance was seen to suppress the production of immune substances. Nutritional support is recognized as one of the most significant aspects of care for the burned patient (11) and soybean peptides and protein can provide an effective way to supplement nutrition. Previous studies have shown that burns cause a significant increase in the WBC counts in rats. WBCs originate from pluripotent hematopoietic stem cells (34, 35). Under the influence of various external stimuli (cytokines, matrix proteins, and accessory cells), stem cells develop into hematopoietic progenitor cells of various lineages (34). Burn injury may causes cytokine release resulting in the production of WBCs. Protein is an important part of a WBC. In this assay, the protein nutrient supplementation significantly increased the number of leukocytes in the blood, peaking 4 days ahead of the burned rats administered with PBS. There was a reduction in the burn injury-induced WBC count increases in protein nutrient supplementation rats compared to those of PBS-treated rats after day 7. These results, therefore, suggest that protein nutrient supplementation could provide early clinical treatment for severe burns, improve the immune stress response rate and ability after the burn, reduce excessive metabolic consumption, maintain organ function, and gain more time for treatment and prognosis. Furthermore, the form of protein supplementation has an effect on WBC production. In this study, the number of leukocytes tended to be higher in the peptides groups than in the intact protein group, indicating that small molecular weight peptides may be the component responsible for the production of leukocytes. Previous studies have found protein-hydrolyzed peptide to be a more digestible and absorbable nitrogen source than protein concentrate. Here, soybean protein was readily digested in the stomach but migrated slowly in the small intestine, indicating that its absorption rate is low (36, 37). Because the soybean peptide has a good equilibrium of essential amino acids and a fast absorption rate, it can provide a better effect on immune stimulation compared with intact protein at an early stage.

In addition, burns were found to affect the appetite of rats, causing a decrease in food intake. Instead, the rats obtained their energy for life activities by decomposing nutrients in their tissues and organs, resulting in the loss of immune organ weight and muscle atrophy. However, the spleen index and thymus index of negative nitrogen rats significantly increased with protein nutrient supplementation. Leukocytes can cause damage to the vascular endothelium by releasing oxygen free radicals and proteolytic enzymes, thus releasing specific thymus-dependent antigens and stimulating the conversion of B cells into plasma cells to release antibodies (38–40). As an important role player in humoral immunity, serum immunoglobulin is the main antibody for mediator fluid immunity. Usually, the immunoglobulins IgM, IgG, and IgA in serum can be used to represent the overall level of serum immunoglobulin (41). The results show that the nutrient supplementation of soybean peptides significantly promoted the formation of serum IgM, IgG, and IgA of negative nitrogen balanced rats after their burn, which could improve the humoral immune function. Protein nutrient supplementation increased the leukocyte production of rats administered soybean peptides and protein on the third day after the burn and accelerated the conversion of B cells into plasma cells to release antibodies, which significantly increased antibody levels. The number of WBC increased rapidly after day 3 of the burn in each group, making no significant difference to their antibody levels. Wang et al. (42) similarly demonstrated that adding 3 g of small peptides per kg BW (body weight) to the basal diets of piglets increased the concentration of immunoglobulin. Feng et al. (43) showed that fermented soybean meal increased the level of serum IgA and IgM in broilers. Soybean peptides may, thus, not only provide nutrients but also synthesize enzymes to promote antibody secretion.

Burn injuries cause major inflammatory reactions and trigger the release of cytokines, which play a vital role in the immune system response against infections and inflammatory diseases. The inflammatory process is central to a number of diseases, so anti-inflammatory peptides are potentially valuable to healing. Nutritional supplementation inhibits the production of major inflammatory cytokines, thereby inhibiting severe inflammatory responses. In this study, soybean peptides and protein significantly suppressed the over-production of IL-1β and TNF-α in the serum of aged rats with a negative nitrogen balance after a burn injury. Young et al. (44) investigated the effect of soy peptide administration on inflammation and inflammatory regulators by examining the protein levels and expressions of key genes involved in the innate immune response as well as the adaptive T-cell response. Concentrations of TNF-α and IL-6 cytokines were found to be lower in the soy peptide treatment and negative control groups than in the dextran sodium sulfate-positive control pigs used in the study. Ndiaye et al. found that protein hydrolysates from soybean seeds could inhibit anti-inflammatory activity by suppressing NO production in activated macrophages as well as suppressing the production of TNF-α. Recent studies have demonstrated that blocking the generation of reactive oxygen species (ROS) leads to decreased NLRP3 inflammasome activity and, subsequently, decreases IL-1β maturation and inflammation. Soybean peptides may also inhibit the expression of NLRP3 through modulating receptors, such as toll-like receptor (TLR)-4/nuclear factor (NF)κB (45).

Burn injuries cause significant changes in amino acid metabolism and plasma amino acids, however, many reparative and immunological functions may be dependent on the availability of certain amino acids. Therefore, protein nutrient supplementation may improve cell-mediated immunity and wound healing. Previous research has shown that glutamine supplementation in burned adults promoted protein synthesis, improved wound healing and shortened their hospital stays (46). As shown in Fig. 7, the presence of exudates were observed in the PBS-treated burn-injured rats after day 11. However, the burn-injured rats that were administered SPP had a better improvement in their peripheral tissue induration than the rats that were administered soybean protein. Nutrient supplementation with SPP may, therefore, be considered to not only decrease the inflammatory response but also improve burn injury outcomes. The normal physiology of aging makes an elderly adult highly susceptible to a poor recovery from burns. Even after hospital discharge and wound closure, muscle breakdown has been observed for up to 9 months after a severe burn (11). Consequently, as an aid to inhibit the muscle breakdown, the administration of soybean peptides may be used to improve body composition and strength in the severely burned aged.

## Conclusion

The results of this study show that soybean peptides mitigated the major full-thickness burn injury-induced inflammatory response in a negative nitrogen balanced aged rat model by modulating the generation speed and ability of systemic WBCs, the immunoglobulin IgM, IgG, and IgA levels, as well as the pro-inflammatory cytokines IL-1β, TNF-α, and RANTES levels. This study provides evidence that nutrient supplementation with soybean peptides could increase the speed of inflammatory stress, decrease the inflammatory response and improve burn injury healing. These results, therefore, suggest that SPP nutrient supplementation offers great potential as a practical and effective intervention for the treatment of severe burn injuries and help to improve the outcomes for these patients. Authors should discuss the results and how they can be interpreted in perspective of previous studies and of the working hypotheses. The findings and their implications should be discussed in the broadest context possible. Future research directions may also be highlighted.
